# Cancer Treatment-Related Infertility: A Critical Review of the Evidence

**DOI:** 10.1093/jncics/pkz008

**Published:** 2019-04-09

**Authors:** Philip D Poorvu, A Lindsay Frazier, Angela M Feraco, Peter E Manley, Elizabeth S Ginsburg, Marc R Laufer, Ann S LaCasce, Lisa R Diller, Ann H Partridge

## Abstract

Cancer treatments may compromise the fertility of children, adolescents, and young adults, and treatment-related infertility represents an important survivorship issue that should be addressed at diagnosis and in follow-up to ensure optimal decision-making, including consideration of pursuing fertility preservation. Risk of infertility varies substantially with patient and treatment factors. The ability to accurately assess fertility risk for many patients is hampered by limitations of the current literature, including heterogeneity in patient populations, treatments, and outcome measures. In this article, we review and synthesize the available data to estimate fertility risks from modern cancer treatments for both children and adult cancer survivors to enable clinicians to counsel patients about future fertility.

Over the past decades, therapeutic advances have transformed the care of cancer patients, yielding substantial improvements in cure rates and survival. Children and young adults have gained particular benefit, given successes realized in the treatment of childhood acute lymphoblastic leukemia (ALL), Hodgkin lymphoma (HL), and testicular cancer, among other malignancies. These gains have come with many costs, and, of the long-term treatment sequelae, infertility is among the most important to patients. The most comprehensive definition of “cancer survivor” includes patients from diagnosis onwards and recognizes that management of survivorship issues, even those arising years after treatment, must start at diagnosis (1). Fertility is a survivorship issue that needs to be discussed before initiating therapy to allow for informed decision-making regarding treatment options, family planning, and fertility preservation strategies (2). Fertility should also be considered throughout follow-up, as needed, to address timing and safety of potential pregnancy. Proactively addressing this critical survivorship issue for those at risk for infertility is associated with lower regret and improved quality of life (3). Thus, clinicians should be able to counsel patients with accurate, up-to-date evidence about this critical issue.

Several recent reviews of fertility preservation strategies have been published (4–7) and guidelines crafted (8–10). However, to our knowledge, there is no recent evaluation regarding the risk of infertility associated with specific diseases and therapies among different age groups. This review synthesizes the literature and summarizes the current best estimates of fertility risk from modern day cancer treatment for children and adults to enable clinicians to counsel patients with the most up-to-date understanding of their risks and the potential indication for fertility preservation.

## Methods

**Figure 1.**
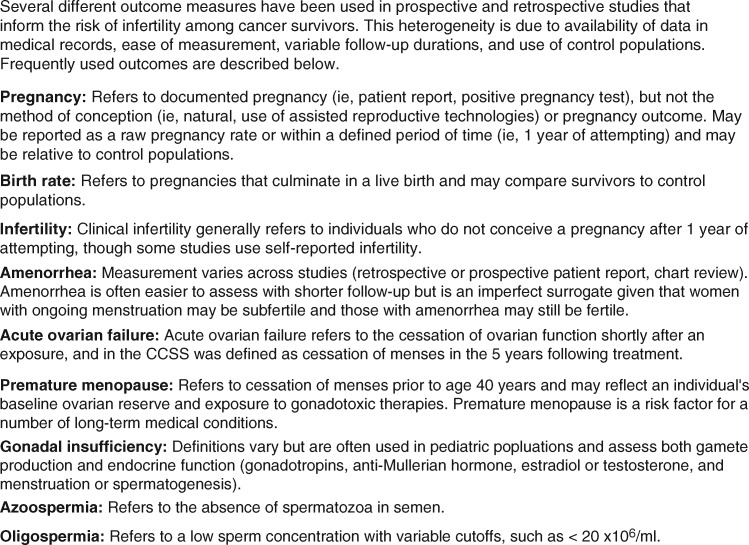
Definitions of fertility-related outcomes.

**Figure 2.**
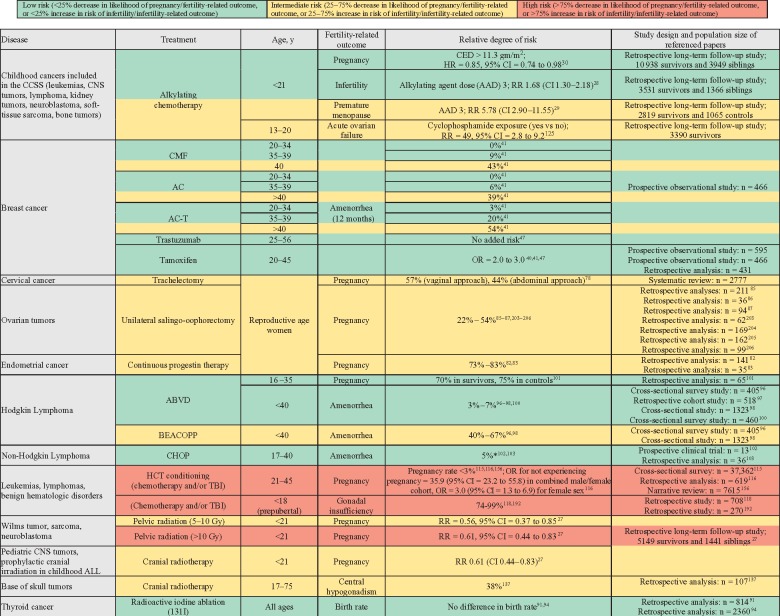
Risks to female fertility associated with cancer treatments. AAD = alkylating agent dose; ABVD = doxorubicin, bleomycin, vinblastine, dacarbazine; AC = doxorubicin, cyclophosphamide; AC-T = doxorubicin, cyclophosphamide, paclitaxel; ALL = acute lymphoblastic leukemia; BEACOPP = bleomycin, etoposide, doxorubicin, cyclophosphamide, vincristine, procarbazine, prednisone; CED = cyclophosphamide equivalent dose; CHOP = cyclophosphamide, doxorubicin, vincristine, prednisone; CI = confidence interval; CMF = cyclophosphamide, methotrexate, fluorouracil; CNS = central nervous system; HCT = hematopoietic cell transplantation; OR = odds ratio; RR = relative risk; TBI = total body irradiation.

**Figure 3.**
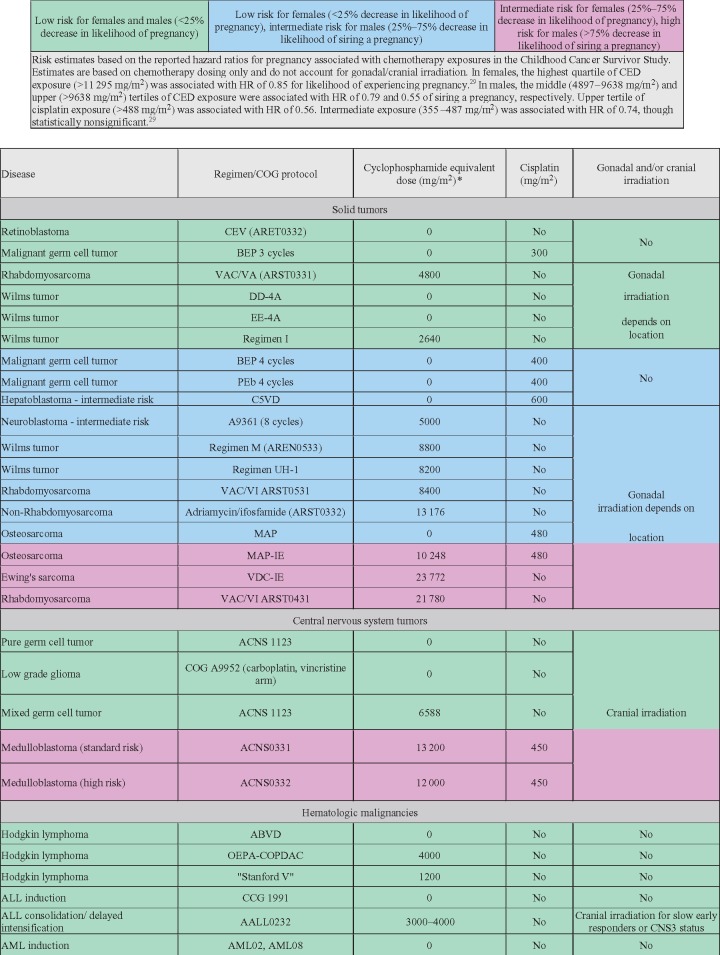
Estimated risk to male and female fertility from select pediatric chemotherapy regimens using calculated cyclophosphamide equivalent dose (CED) and cisplatin exposures. *CED dose (mg/m^2^) = 1.0 (cyclophosphamide dose [mg/m^2^]) + 0.244 (ifosfamide dose [mg/m^2^]) + 0.857 (procarbazine dose [mg/m^2^]) + 14.286 (chlorambucil dose [mg/m^2^]) + 15.0 (carmustine dose [mg/m^2^]) + 16.0 (lomustine dose [mg/m^2^]) + 40 (melphalan dose [mg/m^2^]) + 50 (thiotepa dose [mg/m^2^]) + 100 (nitrogen mustard dose [mg/m^2^]) + 8.823 (busulfan dose [mg/m^2^]). HR = hazard ratio.

**Figure 4.**
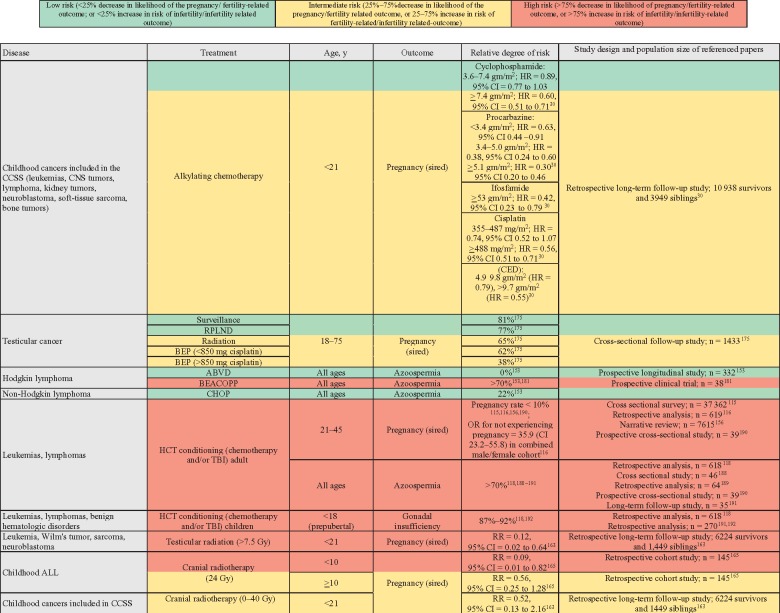
Risks to male fertility associated with cancer treatments. ABVD = doxorubicin, bleomycin, vinblastine, dacarbazine; ALL = acute lymphoblastic leukemia; BEACOPP = bleomycin, etoposide, doxorubicin, cyclophosphamide, vincristine, procarbazine, prednisone; BEP = bleomycin, etoposide, cisplatin; CCSS = Childhood Cancer Survivor Study; CED = cyclophosphamide equivalent dose; CHOP = cyclophosphamide, doxorubicin, vincristine, prednisone; CNS = central nervous system; HCT = hematopoietic cell transplantation; PCI = prophylactic cranial irradiation; RPLND = retroperitoneal lymph node dissection; TBI = total body irradiation.

We performed a review of published articles describing the risks of cancer treatments to female and male fertility using the PubMed database. Search terms included, but were not limited to, names of malignancies (eg, breast cancer, leukemia), cancer therapies (eg, chemotherapies, hormone therapies, biologics, and radiation techniques), and outcomes (eg, pregnancies, birth, fertility, infertility, amenorrhea, azoospermia). We included only peer-reviewed articles written in English. No date cutoff was imposed. Reference lists were reviewed for additional relevant articles. All study designs were considered. Randomized trials and prospective observational studies were included preferentially over retrospective studies. For the purposes of this review, the primary outcome of interest is fertility (the ability to conceive a child), and we thus report pregnancy and live birth data whenever possible and use surrogates, such as amenorrhea, semen parameters, and laboratory markers of gonadal insufficiency, as alternatives when necessary (Figure 1). Findings are broken down by sex and age group (pediatric vs adult) and by cancer type for ease of reference. They are further grouped by general degree of risk (high, intermediate, and low) as detailed in Figures 2–4 and color-coded.

## Female Fertility

Data from prior prospective and retrospective trials inform the risks of infertility, yet limitations exist in assessing the likelihood that an individual patient will remain fertile. Ovarian reserve varies among women and is affected by additional factors, including genetic polymorphisms associated with age of menopause (11) and others in genes encoding drug metabolism enzymes (12, 13) may also affect risk of ovarian toxicity. Psychosocial factors, many of which are affected by cancer diagnosis and treatment, are also important determinants of reproductive choices and may have important implications when comparing fecundity of survivors to control populations (14).

Due to ease of measurement, amenorrhea is frequently reported as a primary outcome yet is an imperfect predictor of fertility. Female survivors who have spontaneous menses, particularly if irregular, may still have decreased ovarian reserve and reproductive potential (15–19). Abnormalities of traditional laboratory markers such as follicle-stimulating hormone, estradiol, and inhibin-B levels are late markers of ovarian aging (20). Anti-Mullerian hormone (AMH) is more strongly correlated with antral follicle count (21) and is an earlier predictor of decreased ovarian reserve (22). AMH is evaluable in both pre- and postmenarchal females (23) and detects diminished ovarian reserve among female cancer survivors (17, 18, 24, 25). AMH may therefore be a preferred laboratory assay for establishing a pretreatment baseline, assessing ovarian reserve among survivors, and determining who might benefit from fertility preservation services (26).

The following section details data regarding fertility risks in select populations, diseases, and treatments and is presented with a summary in Figure 2.

### Childhood Cancers

Multiple reports from the Childhood Cancer Survivor Study (CCSS), a multi-institutional long-term follow-up study that included patients and matched sibling control subjects, have demonstrated associations between chemotherapy use, as measured by summed alkylating agent dose or cyclophosphamide equivalent dose (CED), and risk of clinical infertility, never achieving pregnancy, and premature menopause (27–30). A 2016 CCSS report, which included 10 938 patients treated between 1970 and 1999 and unexposed to pelvic or brain radiation, found that alkylating chemotherapy decreased the likelihood of pregnancy only at the highest quartile of CED exposure (>11.3 gm/m^2^, hazard ratio [HR] = 0.85, 95% CI = 0.74 to 0.98) , suggesting that most survivors treated with a variety of chemotherapy regimens alone are not at high risk of infertility (Figure 3) (30). Most studies support the impact of alkylating chemotherapy on infertility and surrogates, including acute ovarian failure (AOF) and premature menopause (19, 31–33), but not all have identified a detrimental effect (34, 35). The 2016 CCSS report did not identify an association between platinum agents (cisplatin, carboplatin) and the likelihood of pregnancy in female childhood cancer survivors (30). However, females with germ cell tumors were not included, and whether platinum administered in this setting (following unilateral oophorectomy) affects future fertility remains unknown. The CCSS represents the most robust source of data, and considering the body of literature, there is strong evidence that higher levels of exposure to alkylating chemotherapy have a greater effect on fertility and biological surrogates. Although the absence of statistical significance with lower exposures in CCSS is reassuring, modern day regimens that include compressed chemotherapy and/or higher CED levels have not been fully evaluated and it is therefore reasonable to use the lowest alkylator dose possible that is associated with the best cure rates (36).

Patient and disease factors appear to have little impact upon subsequent fertility outcomes among children. Age at diagnosis is an important predictor of fertility-related outcomes in some adult malignancies; however, data from the CCSS showed no association between age at diagnosis and future fertility (30). Although HL has been found to be an independent risk factor for premature menopause and is associated with diminished pretreatment ovarian reserve in adult women (29, 37), a large prospective cohort study of female childhood HL survivors found that rates of parenthood were comparable to the German general population through age 39 years, suggesting that the impact of HL and its treatment on fertility is not substantial until the majority of the reproductive years have passed (38). Furthermore, regimens that include alkylating chemotherapy, which are associated with lower AMH values relative to doxorubicin, bleomycin, vinblastine, dacarbazine (ABVD) (25), are being used less frequently in modern therapy, limiting the impact upon fertility.

### Breast Cancer

Among adults, the most robust data regarding fertility risks are available for women with breast cancer. In a recent meta-analysis of five prospective, randomized trials evaluating gonadotropin-release hormone (GnRH) agonists during breast cancer chemotherapy, the rate of premature ovarian insufficiency in the control (chemotherapy alone) arms was 31%, demonstrating that many premenopausal women retain ovarian function (39). Importantly, older age is a well-established risk factor for treatment-related amenorrhea (TRA) following alkylating chemotherapy for breast cancer (40–47), with rates of 0–15%, 30%–50%, and about 70% among women age younger than 35 years, 36–40 years, and older than 40 years, respectively, and younger women who experience TRA are more likely to resume menses (40, 41, 47). Thus, when considering absolute rates of TRA, the age distribution of study populations must be considered.

Cyclophosphamide, included in most adjuvant regimens, appears to be the primary driver of TRA. In a randomized trial comparing the efficacy of a doxorubicin/docetaxel to regimens combining cyclophosphamide, doxorubicin, and a taxane, either sequentially (AC-T) or concurrently (TAC), in which 70% of participants were older than 40 years old, rates of TRA were lower with doxorubicin/docetaxel (38%) relative to AC-T (70%) and TAC (58%) (*P* < .001) (48). How much cyclophosphamide and how it is administered also appears to contribute to heterogeneity in early and late surrogates of ovarian toxicity. A prospective study of menstrual patterns, in which 46% of participants were over 40 years of age, revealed monthly bleeding immediately following chemotherapy in only 16% of premenopausal women treated with AC-based regimens, in which cyclophosphamide is delivered in four intravenous infusions, vs 48% with CMF, in which cyclophosphamide is taken orally daily for 14 days of six 28-day cycles (40). However, the proportion with monthly bleeding 24 months following chemotherapy rose to 50% among those who received AC, but declined to 30% among those treated with CMF.

Additional data inform the risks of TRA with taxanes, which are now frequently included in adjuvant regimens. AC-T has been associated with numerically higher rates of TRA than AC alone (29% vs 19% in a high-quality prospective study and an odds ratio [OR] of 1.59 [95% CI = 0.8 to 3.2] in a large retrospective study), though neither difference was statistically significant (41, 47). In a study of patients with small HER2+ tumors treated with paclitaxel/trastuzumab, TRA occurred in 18 of 64 (28%) premenopausal women, but in only 1 of 11 (9%) women age 40 years or younger vs 14 of 29 (48%) women aged over 45 years (49). Data on docetaxel/cyclophosphamide is limited to one prospective cohort study of women age 40 years and younger in which the rate of 12-month TRA with docetaxel/cyclophosphamide was 33% and similar to AC (46%) and AC-T (40%) (50). One study suggested that docetaxel could be more gonadotoxic than paclitaxel; AC followed by docetaxel was associated with statistically significantly higher risk of TRA than AC (OR = 9.4) and AC-T (OR = 7.2), though conclusions are limited by the small number of patients treated with AC followed by docetaxel (n = 17) (41). Although some reports have identified taxanes as an independent risk factor (41, 46), most have not (42, 43, 45, 51–54). Recognizing limitations in the literature, especially studies grouping paclitaxel and docetaxel, the available data suggest taxanes may contribute to TRA, but that the absolute effect appears to be small and the predominant predictors of amenorrhea for women receiving taxane-containing chemotherapy are age and exposure to alkylating chemotherapy.

Neither dose-dense schedule nor the addition of trastuzumab has been associated with TRA (47, 55). Platinum agents are increasingly being explored for triple negative breast cancer and it will be important to characterize their impact on TRA. Cisplatin, a DNA cross-linking agent, appears gonadotoxic in some settings, though published data are scarce (56, 57). Cisplatin was associated with reduced pregnancy rates in males, but not females, in the CCSS (30, 57, 58). In one small study of women diagnosed at age 45 years or younger with breast cancer (n = 165), the rate of 12-month TRA was only 6% among the 35 women treated with carboplatin-docetaxel (TP) (59). Given the limited data and plausible mechanism of gonadotoxicity, it is most appropriate to counsel patients that platinum agents, particularly cisplatin, may impair fertility.

Tamoxifen use following adjuvant chemotherapy is associated with a 2-fold increase in risk of TRA (40, 41, 47, 60), though no women age 40 years or younger treated with tamoxifen alone developed amenorrhea within a large prospective cohort study (50). Tamoxifen does not appear to have permanent effects on menstrual function or fertility but is teratogenic and must be held before and during pregnancy; thus, the need to delay childbearing can pose a risk to fertility through ovarian aging.

TRA has been associated with improved overall survival in breast cancer (61); thus, reversible means of obtaining this “chemoendocrine effect” may be desirable for women interested in future fertility. Ovarian suppression with GnRH agonists is increasingly being incorporated into the treatment of premenopausal women with hormone-sensitive disease (62, 63). Although chemotherapy has often been the default approach for young women, optimization of endocrine therapy with ovarian suppression may be a more prudent approach for some with lower risk disease and a means of preserving future fertility.

### Gynecologic Malignancies

In the treatment of cervical cancer, the focus for women interested in future fertility has been on prevention of anatomical changes that impair childbearing. The risk of infertility with hysterectomy is 100%, although successful pregnancies have occurred with oocyte retrieval and use of a surrogate (64–66). Fertility-sparing procedures, such as vaginal or abdominal radical trachelectomy in which the cervix and upper vagina are removed, are now options for highly selected patients (67). Pregnancy rates among women attempting to conceive range from 25% to 95%, with most estimates above 40% (68–77). Whereas all women in one series required in vitro fertilization (IVF) after treatment, 16 of 17 patients who conceived in another series did so naturally (68, 75). Miscarriage rates range from 9% to 42% and rates of pregnancies with gestation beyond 37 weeks range from 14% to 55% (69–74). A recent systematic review found improved pregnancy rates associated with vaginal radical trachelectomy (57%) vs laparotomic abdominal radical trachelectomy (44%) (78). Candidates for fertility-sparing procedures generally do not receive adjuvant radiation or chemotherapy (67). However, neoadjuvant chemotherapy (cisplatin plus ifosfamide in squamous cell carcinoma or cisplatin plus doxorubicin in adenocarcinoma) has been used on an experimental basis to downstage patients who were not upfront candidates for fertility-sparing surgery and resulted in fertility preservation in 20 of 28 patients (71%), 10 of whom (50%) became pregnant in one series (79). Pregnancy data are not available for women receiving definitive concurrent chemoradiation. A small series demonstrated the feasibility of ovarian transposition before chemoradiation, with ovarian failure experienced by 1 of 7 women age 40 years or younger and 6 of 7 women aged over 40 years (80).

Although hysterectomy with bilateral salpingo-oophorectomy represents the standard approach to localized endometrial cancer, continuous progestin therapy is a fertility-sparing option for highly selected young women with endometrial hyperplasia or stage IA endometrial adenocarcinoma (81). Although reproductive outcomes data are limited, the two largest series demonstrated pregnancies in 51 of 70 patients (73%) and 10 of 12 patients (83%) who attempted pregnancy after achieving a complete remission (82, 83).

Management of epithelial ovarian cancer also includes removal of critical reproductive organs. For highly selected women, depending on extent and type of disease, fertility-sparing procedures with unilateral salpingo-oophorectomy and complete surgical staging may be performed (84). In one study of women with unilateral stage I invasive epithelial ovarian cancer, 182 of 186 women (96.8%) remained premenopausal postoperatively (85). Pregnancy rates among women attempting to conceive after unilateral salpingo-oophorectomy for EOC have ranged from 27% to 53% (85–88).

Gonadal-sparing surgery is the goal of management of ovarian cyst and tumors in adolescent and young adult women. Most ovarian tumors in girls and adolescents are benign. Malignant tumors are usually of germ cell origin. For tumor marker negative tumors, a fertility-sparing procedure can be performed. With positive tumor markers, a unilateral oophorectomy and staging procedure—in which peritoneal fluid is collected, lymph nodes and omentum are inspected, and biopsy reserved for suspicious sites—is performed to maintain fertility. Minimizing abdominopelvic surgery prevents fertility issues due to adhesions. Most ovarian germ cell tumors in adolescents and young adults are stage I and about 50% are cured with surgery. For women requiring further treatment, fertility preservation should be considered, given the effect of cisplatin on ovarian function remains unknown in this setting.

### Thyroid Cancer

Thyroidectomy represents the primary treatment for localized, differentiated thyroid cancer and, with thyroid hormone replacement, is not gonadotoxic. Radioactive iodine ablation (^131^I) may also be used for differentiated thyroid cancer and leads to transient amenorrhea in up to 20% of patients (89). The average age of menopause is slightly lower following ^131^I (49.5 vs 51.0 years), but there is no difference in birth rate and ^131^I does not appear to have long-term effects on fertility (90–94).

### Hodgkin Lymphoma

The majority of adult women diagnosed with HL are of reproductive age and, given their excellent cancer prognosis, fertility in survivorship may be particularly important (95). A robust literature demonstrates that TRA among HL survivors is affected by age, systemic therapy, and exposure to pelvic radiation (16, 96, 97). Rates of TRA are high among regimens that contain heavy alkylator exposure: cyclophosphamide, vincristine, procarbazine, prednisone, doxorubicin, bleomycin, vinblastine, dacarbazine (COPP/ABVD) (54.6%), bleomycin, etoposide, doxorubicin, cyclophosphamide, vincristine, procarbazine, prednison (BEACOPP) (47%–56%), and dose-escalated BEACOPP (40%–67%) (96–98). ABVD is the standard initial chemotherapy in the United States for adults and is associated with very low rates of TRA (3%–7%) (96–100). Pregnancy rates following ABVD are similar to control subjects (70% vs 75%) (101). Thus, fertility preservation is usually not needed for women receiving ABVD alone. Patients are often instructed to delay pregnancy for 2 years beyond treatment (when most relapses occur); thus, fertility preservation measures could be considered if this delay would substantially reduce the remaining fertile period. Ongoing trials integrating targeted and immune-based therapies such as brentuximab and nivolumab into upfront HL therapy, both of which are unlikely to directly affect fertility, may further limit the need for gonadotoxic alkylating chemotherapy.

### Non-Hodgkin Lymphoma

Non-Hodgkin lymphoma (NHL) encompasses indolent and aggressive diseases addressed with a variety of treatments, including chemotherapy, targeted therapies, and immune-based therapies. Some studies suggest relatively low fertility rates for female survivors, with successful pregnancies in 21% (15) and TRA in 41% (16). The total cyclophosphamide exposure with six cycles of cyclophosphamide, doxorubicin, vincristine, prednisone (CHOP; 4500 mg/m^2^) is nearly twice that of adjuvant breast cancer regimens, and therefore the effects on amenorrhea and fertility are expected to be at least that of women of corresponding age treated for breast cancer. However, young women with breast cancer have low rates of amenorrhea, and other studies suggest more favorable fertility outcomes for NHL survivors with greater than 50% achieving pregnancy and TRA in under 10%, even with dose-intensified CHOP (cyclophosphamide 12 000 mg/m^2^) (102, 103). Older women are more likely to experience AOF (15). Women treated with rituximab, an anti-CD20 monoclonal antibody, are counseled to avoid pregnancy given concerns regarding teratogenicity and immunosuppression of offspring (104). No data are available regarding the impact of rituximab on female fertility, though the mechanism of action would not be expected to directly affect fertility. Treatment of relapsed NHL (and HL) with chemotherapy or autologous hematopoietic cell transplantation (HCT) is clearly associated with worse reproductive outcomes due largely to greater use of alkylator chemotherapy.

### Leukemia

The backbone treatment for acute myeloid leukemia (other than acute promyelocytic leukemia) remains daunorubicin, an anthracycline, and cytarabine. Fertility risks associated with anthracyclines are poorly defined because anthracyclines have usually been co-administered with alkylators for more prevalent cancers. However, rates of amenorrhea with anthracyclines may be substantial even in the absence of alkylators (48). Treatment of ALL often includes alkylating agents and risk presumably varies by the regimen selected. Limited studies on menstrual function and fertility, often grouping patients with AML and ALL, have demonstrated rates of AOF with induction chemotherapy as low as 17% (15, 105), though AOF may underrepresent the impact of treatment on fertility (15). The greatest threat to fertility among women with leukemia is gonadotoxic conditioning before HCT, as exhibited by lower rates of infertility among women who receive consolidation chemotherapy (106). The urgency to initiate induction therapy generally precludes standard fertility preservation measures for women with acute leukemia, but opportunities may exist in the first complete remission or before induction therapy for those with high-risk myelodysplasia, especially because pregnancies can be achieved after HCT with banked embryos or oocytes (107).

The risk to fertility associated with long-term imatinib, a tyrosine kinase inhibitor (TKI) targeting bcr-abl, for chronic myeloid leukemia is not well studied. Offspring of women exposed to imatinib during pregnancy have demonstrated congenital abnormalities, and patients should practice reliable contraception while on TKI therapy, including imatinib or others (108–110). Despite case reports of impaired ovarian function (111) and premature ovarian failure (112), it is not clear whether imatinib or other TKIs affect fertility. Patients who discontinue are at risk for loss of response (113); thus, the benefit of continuing therapy during reproductive years represents a threat to fertility and consideration can be given to alternative strategies for having future biologic children, including use of a gestational carrier.

### Hematopoietic Cell Transplant

Survivors who undergo HCT are at high risk for ovarian failure and infertility due to the treatment of their primary disease and the conditioning regimen, which generally includes gonadotoxic chemotherapy with or without radiation. However, many pregnancies have been identified following HCT, including autologous and nonmyeloablative and myeloablative allogeneic transplants for malignant and nonmalignant conditions (114–116). True risks of infertility and amenorrhea have been difficult to assess given heterogeneous patient populations and that not all survivors desire or attempt pregnancy (117). Of 708 postmenarchal women in one study, 110 (16%) recovered ovarian function and 32 (5%) subsequently became pregnant (118). Of 82 premenarchal girls, 23 (28%) developed normal gonadal function and 9 (11%) became pregnant (118). In another study of HCT survivors with a median age of 33 years and follow-up of 8 years, 8 of 292 (3%) female survivors became pregnant vs 72% of sibling control subjects (116). In the entire cohort (including men), survivors were dramatically less likely to report pregnancy (OR = 35.9, 95% CI = 23.2 to 55.8). Female sex was an independent risk factor for infertility among survivors (OR = 3.0, 95% CI = 1.3 to 6.9), though use of assisted reproductive technologies (ART), which may be have been more common among men given availability of sperm banking, was not recorded (116). Pregnancy rates were low following allogeneic and autologous HCT, and predictors of infertility included age 30 years or older (OR = 4.8, 95% CI = 2.1 to 10.7) and receipt of total body irradiation (TBI) (OR = 3.3, 95% CI = 1.5 to 7.3) (116). Although one prospective cohort study of HCT survivors with an average age at diagnosis of 33 years identified no pregnancies among 60 females 10 years after HCT, other studies have demonstrated pregnancy rates of 40%–65% and regular menstrual cycles in 63%–68% among autologous HCT survivors under age 40 years (119–121). One early prospective study demonstrated that regular menses resumed in only 6% of women transplanted for leukemia with cyclophosphamide and TBI conditioning regimens but in 74% of those transplanted for aplastic anemia (and 100% age <26 years) with cyclophosphamide only conditioning regimens, and both increasing age at diagnosis (relative risk [RR]_per year_ 1.2, *P* = .002) and receipt of TBI (RR_12 Gy vs none_ 8.3, *P* < .001) were associated with ovarian failure (122). Thus, although fertility may be severely impaired by transplant, risk varies considerably by age and conditioning regimen. All females undergoing HCT should be assumed to be at intermediate to high risk of ovarian failure and infertility, and additional research is needed to help risk-stratify women based on patient, disease, and treatment characteristics.

### Radiotherapy

Radiation can diminish fertility when directed to reproductive organs or the structures that produce hormones necessary for reproduction. Mathematical modeling suggests the dose at which 50% of immature oocytes die is under 2 Gy (123). The exposure required to induce ovarian failure and infertility decreases with increased age, due to the normal decline in ovarian reserve and an increase in radiosensitivity of oocytes in growing follicles relative to primordial oocytes (124–127).

The CCSS demonstrated a dose-dependent effect of radiation on risk of ever being pregnant (5–10 Gy, RR = 0.56, 95% CI = 0.37 to 0.85; >10 Gy, RR = 0.18, 95% CI = 0.13 to 0.26) relative to sibling control subjects (27). Radiation exposures as low as 1 Gy are associated with increased risk of premature menopause (RR = 4.3, 95% CI = 1.2 to 15.5) and AOF (RR = 3.6, 95% CI = 1.9 to 7.2) (29, 125). More than 80% of girls with exposure greater than 20 Gy experienced AOF (125). TRA occurred in all 11 women within the Georgia Cancer Registry exposed to pelvic radiation for a variety of malignancies (16). A retrospective series also demonstrated TRA in only 5% of premenopausal women treated for colon cancer, but in 94% treated for rectal cancer, likely due at least in part to pelvic radiation (128). Scattered radiation from abdominal fields may reach the ovary, with one study showing a median exposure of 1 Gy, and should be considered when assessing the infertility risk from abdominal radiation (129).

Pelvic radiation may also affect the uterus, leading to atrophy of the myometrium and endometrium (130) and decreased uterine length and blood flow (131, 132). The degree to which uterine effects contribute is unclear, though patients treated with pelvic radiation are known to be at substantial risk for pregnancy complications (115).

Cranial irradiation affects fertility through the development of endocrinopathies involving the hypothalamic-pituitary-gonadal axis (133). Among children (male and female), prophylactic cranial irradiation (PCI) (18–24 Gy) for ALL is associated with a lower birth rate than chemotherapy alone (134, 135). The CCSS confirmed that hypothalamic/pituitary radiation doses of 22–27 Gy (HR = 0.67, 95% CI = 0.53 to 0.84) and over 30 Gy (RR = 0.61, 95% CI = 0.44 to 0.83) are associated with decreased fertility among females (27, 136). Whether the decreased radiation doses currently used for PCI and implementation of proton radiotherapy for CNS malignancies will mitigate fertility risk remains unknown (133). The 5-year risk of central hypogonadism in one study of patients treated with conformal radiation techniques for base of skull tumors was 38% among females, with onset ranging from 2 to 11 years after treatment (137); thus, survivors remain at risk for infertility years beyond diagnosis.

## Male Fertility

Infertility among male cancer survivors is common yet has been the subject of less research than female infertility. Pregnancy and live birth data are sparse, and spermatogenesis parameters are frequently used as surrogates although generally felt to be more reliable than markers of ovarian reserve. Semen analysis methodology and reference ranges for semen volume, sperm concentration, total sperm number, morphology, and motility have been standardized (138, 139). Each parameter has been associated with time to pregnancy and/or probability of conception (139–143). Today, use of in vitro fertilization and intracytoplasmic sperm injection overcome most sperm defects and allow successful pregnancy. Semen analysis does not assess the cellular and biochemical processes required to bind, penetrate, and fertilize the oocyte (139) or genetic contributors to infertility (144); thus, a normal semen analysis does not guarantee fertility.

Although treatment usually represents the greatest threat to fertility of male survivors, the underlying malignancy may affect semen production and quality through poorly understood mechanisms. In testicular cancer, rates of oligospermia and azoospermia at presentation are 50% and 10%, respectively (145–148). Similarly, HL is associated with oligospermia and azoospermia before treatment (149–151). Interestingly, treatment of the underlying malignancy may yield improvements in spermatogenesis (152–155).

Radiation and chemotherapy have cytotoxic effects on testicular germinal epithelium including Sertoli cells, but to a lesser extent on Leydig cells, leading to frequent impairment of spermatogenesis without hypogonadism (117, 156). Sperm counts fall dramatically within 2 months following chemotherapy or radiation (157). Lack of early recovery does not necessarily portend permanent sterility. Recovery of spermatogenesis can occur up to 5 years after treatment (158), and spermatozoa have been successfully extracted via microdissection testicular sperm extraction in up to 37% of azoospermic males in whom it was attempted following chemotherapy in one series and 47% in another (159, 160).

The next section details data regarding the risk to male infertility in select populations, diseases, and associated treatment modalities and is presented with a summary in Figure 4.

### Childhood Cancers

Within the CCSS, male survivors were statistically significantly less likely than sibling control subjects to sire a pregnancy, and chemotherapy appears to be a greater risk to fertility for males (HR = 0.63, 95% CI = 0.58 to 0.68) than females (HR = 0.87, 95% CI = 0.81 to 0.94) (30). Among males unexposed to radiation, exposure to individual alkylators, including cyclophosphamide (3.6–7.4 gm/m^2^, HR = 0.89, 95% CI = 0.77 to 1.03; >7.4 gm/m^2^, HR = 0.60, 95% CI = 0.51 to 0.71), ifosfamide (26–53 gm/m^2^, HR = 0.61, 95% CI = 0.36 to 1.01; >53 gm/m^2^, HR = 0.42, 95% CI = 0.23 to 0.79), and procarbazine (<3.3 gm/m^2^, HR = 0.63, 95% CI = 0.44 to 0.91; 3.3–5 gm/m^2^, HR = 0.38, 95% CI = 0.24 to 0.60; >5 gm/m^2^, HR = 0.30, 95% CI = 0.20 to 0.46), was associated with statistically significant decreases in male fecundity. Similar findings were demonstrated when analyzing by CED per 5-gm/m^2^ increment (HR = 0.82, 95% CI = 0.79 to 0.86). Although lacking statistical significance, potentially due to inadequate statistical power, low or moderate exposure to the other alkylators, including busulfan (<450 mg/m^2^, HR = 0.46, 95% CI = 0.15 to 1.42) and lomustine (>411 mg/m^2^, HR = 0.82, 95% CI = 0.26 to 2.60), may still have clinically significant effects on male fertility. This builds upon prior evidence for a dose-dependent increase in risk of infertility and impairment in spermatogenesis among male cancer survivors treated with alkylating chemotherapy (161–164). Among a cohort treated with alkylating chemotherapy and without radiation, 25% were azoospermic and 28% oligospermic, and CED was associated with azoospermia (OR = 1.22 per 1000 mg/m^2^, 95% CI = 1.11 to 1.34) (161). Importantly, the CCSS confirmed that cisplatin, a DNA crosslinking agent, is associated with decreased male fertility (<355 mg/m^2^, HR = 0.85, 95% CI = 0.56 to 1.27; 355–487 mg/m^2^, HR = 0.74, 95% CI = 0.52 to 1.07; > 488 mg/m^2^; HR = 0.56, 95% CI = 0.39 to 0.82) (30). Compared with sibling control subjects, male childhood ALL survivors appear to have normal fertility, presumably due to low alkylator exposure (165). Although early reports suggested that pubertal males are more susceptible to gonadotoxicity, the CCSS and St. Jude’s cohorts did not identify age as a risk factor among males (161, 163, 166).

### Testicular Cancer

Although unilateral orchiectomy theoretically spares the unaffected testis, sperm concentration decreases post-procedure and azoospermia may occur among men who initially presented with oligospermia (167). Treatment of bilateral testicular cancer with bilateral orchiectomy yields sterility, but this presentation represents only 1% of testicular cancers and the majority are metachronous rather than synchronous (168). Retrograde ejaculation was previously a frequent complication of retroperitoneal lymph node dissection (169,170), but is now uncommon with nerve-sparing techniques (171–173).

Despite data suggesting that testicular cancer survivors are as likely to sire a pregnancy as age-matched control subjects (174), adjuvant therapy for testicular cancer affects fecundity in some patients. A prospective study of men treated with unilateral orchiectomy followed by surveillance or adjuvant therapy found that paternity rates without cryopreserved sperm were lower among men who received radiotherapy (65%) or cisplatin (<850 mg [62%]; >850 mg [38%]) than men who underwent surveillance (81%), but no difference was seen between retroperitoneal lymph node dissection (77%) and surveillance (175). Retrograde ejaculation was reported by 54 of 520 men (10%), 12 (22%) of whom conceived without cryopreserved sperm. Subsequent analyses of men receiving adjuvant chemotherapy demonstrated a dose-dependent effect, with post-treatment paternity rates of 100%, 83%, and 76% among men receiving two, three, and four cycles, respectively (176). Similarly, mean sperm counts recover as early as 12 months following two or fewer cycles and by 24 months following more than two cycles, and more men who receive high-dose cisplatin experience prolonged azoospermia (177, 178). Return to normospermia appears to be statistically significantly more likely with carboplatin than cisplatin (HR = 4.5, 95% CI = 2.6 to 7.8) (178).

### Thyroid Cancer

^131^I is associated with a testicular radiation exposure of about 0.10 Gy. No data regarding effects on spermatogenesis or fertility are available (179). Given the gonadotoxicity of radiation doses less than 1 Gy (see Radiotherapy section below), fertility interest should be assessed routinely and sperm banking is prudent for patients interested in future fertility.

### Hodgkin and Non-Hodgkin Lymphoma

Most male HL survivors will have normal fertility following treatment. Within a case-control study, 29.3% of survivors had biological children post-treatment relative to 32.4% of age-matched control subjects (OR = 0.84, 95% CI = 0.70 to 1.00) (14). Among 345 male survivors who attempted pregnancy, 217 (63%) conceived naturally and 49 (15%) with ART. Second line treatment (OR = 0.10, 95% CI = 0.04 to 0.24) and age older than 35 years (OR = 0.16, 95% CI = 0.07 to 0.36) were associated with lower fecundity. Alkylating chemotherapy was associated with a dose-dependent effect (OR = 0.34, 95% CI = 0.16 to 0.70 for <3 MOPP-equivalent cycles; OR = 0.04, 95% CI = 0.02 to 0.09 for >3 MOPP-equivalent cycles).

Over the past several years, therapy has shifted away from regimens heavy in alkylator exposure and towards ABVD. A prospective study that included HL and NHL found that no patients treated with ABVD were azoospermic 1 year post-treatment, with higher rates following CHOP (22%) and BEACOPP (75%) (153). Two years after treatment, sperm counts recovered to normal in 90% following ABVD and only 61% following CHOP. Other studies support the low rate of azoospermia with ABVD (4%) and high rates with BEACOPP (89%) (180–182). The variability in risk between regimens is substantial. Most adult HL patients in the United States are now treated with ABVD and their risk of infertility is low, whereas risk is intermediate or high for those receiving BEACOPP and regimens for aggressive NHL. All men should be encouraged to bank sperm at diagnosis, but opportunities to bank may still exist in early survivorship or in a relapsed setting before re-initiating therapy.

### Leukemia

Limited data are available on risks to fertility among men receiving chemotherapy for acute leukemias. Patients with AML and ALL have been grouped together, despite differences in treatments, including in use of alkylating agents. One series of 13 patients with acute leukemias reported azoospermia in 46% (183), and two of five patients in another series treated with daunarubicin and cytarabine developed severe oligospermia (184). It seems likely that some patients experience at least transient azoospermia, even if some retain or recover spermatogenesis (185). Given the uncertainty, all male patients should be offered sperm banking before treatment, particularly given the effect of HCT on fertility.

Pregnancies have been identified in female partners of males on imatinib therapy for chronic myeloid leukemia, though data are insufficient to conclude whether imatinib is associated with congenital abnormalities in offspring of males (108). The risk of age-related loss of fertility due to delayed conception is less statistically significant for males because interrupting treatment for conception may be more feasible for males than females as only a washout period before conception might be recommended, whereas females are recommended to also hold treatment through the duration of a pregnancy. Although one study used a washout period of 1 month from imatinib (186), spermatogenesis occurs over an estimated 74 days and the minimum safe duration remains unknown (187).

### Hematopoietic Cell Transplantation

Conditioning chemotherapy and radiation frequently lead to severe impairment of male fertility. Fewer than 30% of men recover spermatogenesis and normal testicular function following HCT, with the lower rates of recovery with greater gonadotoxic exposures (118, 188–192). The birth rate among male HCT survivors is statistically significantly lower than the general population, though pregnancies following heavily gonadotoxic conditioning regimens such as BuCy or BEAM and TBI have been reported (114–116, 156). In a retrospective study of 327 adult male HCT survivors with median follow-up of 7.7 years, 26 (8%) had sired a pregnancy, with similar rates following allogeneic and autologous transplants (116). Despite very low pregnancy rates, risk appears to vary by exposures. TBI, older age (>30 years), and chronic GVHD have been associated with impaired spermatogenesis (116, 117, 190). Higher rates of detectable sperm have been found following lower dose BuCy (cyclophosphamide 120 mg/kg) than conventional dosing (cyclophosphamide 200 mg/kg) (81% vs <20) (118, 193). Because no patient is at low risk, sperm banking should be recommended for all pubertal males before HCT.

### Radiotherapy

The testes are sensitive to small radiation exposures, and the effect on spermatogenesis depends upon gonadal dose and radiation schedule (194). Oligospermia occurs following greater than 1 Gy of testicular radiation, and exposures of 4–6 Gy yield profound impairments in spermatogenesis (195). The testes may be exposed to dose scatter in abdominal and pelvic radiation for rectal cancer, testicular cancer, and other malignancies. Abdominal radiation following unilateral orchiectomy for testis cancer yielded exposure to the remaining testicle of 0.09 and 0.32 Gy with paraaortic and dog-leg fields, respectively, in one series, although testicular shields may reduce exposure (196, 197). Among men treated with radiation (0.28–0.9 Gy) following unilateral orchiectomy for testicular seminoma, sperm counts recover following treatment, most within 1 year (148). Radiation therapy for HL may be associated with testicular exposure of 0.06–0.7 Gy and lead to transient oligospermia, but spermatogenesis appears to normalize within 18 months (198). Similarly, among men treated for rectal cancer, exposures under 1.3 Gy lead to transient azoospermia in greater than 70%, but almost all patients recover spermatogenesis (199).

Gonadal exposure of 10 Gy in TBI yields azoospermia in more than 80% of patients, though concurrent alkylating chemotherapy may also contribute (118). Male childhood cancer survivors who receive more than 7.5 Gy are profoundly less likely to sire a pregnancy (HR = 0.12, 95% CI = 0.02 to 0.64), and one study found no pregnancies following more than 10 Gy for childhood ALL (163, 200).

High-dose PCI (>24 Gy) is associated with diminished fertility among adult male survivors of childhood ALL. One analysis demonstrated impaired fertility for survivors treated with 24 Gy at ages younger than 10 years (RR = 0.09, 95% CI = 0.01 to 0.82) and 10 years or older (RR = 0.54, 95% CI = 0.25 to 1.28) (165). Within the CCSS, a statistically nonsignificant trend was identified following 0–40 Gy of hypothalamic/pituitary radiation (HR = 0.52, 95% CI = 0.13 to 2.16) and more than 40 Gy (HR = 0.29, 95% CI = 0.06 to 1.28) (163). Modern protocols now tailor PCI use to risk (CNS leukemia, slower early responders) and use lower doses of 12–18 Gy, which do not appear to affect spermatogenesis (201, 202).

## Conclusion

Young cancer survivors face wide variation in fertility risk attributable to age at diagnosis, disease, and treatment. Nevertheless, there is a need for additional biomarkers to improve prediction of impaired fertility, with emerging data suggesting that measures of ovarian reserve such as AMH may add value. Future studies will need to assess the risks of modern treatment regimens, including potential impact of targeted and immune-based therapies, and the role of ART on pregnancy rates in survivor populations. Recognizing the importance of survivorship issues, clinical trials should aim to incorporate patient-reported outcome measures to collect long-term fertility data. Understanding what is known and what is unknown about fertility risks is needed in order to counsel patients optimally regarding situations in which fertility preservation strategies may be needed and when patients can feel confident foregoing them. Newly diagnosed young patients with cancer and survivors may also benefit from the development and incorporation of counseling tools and guidelines for referral to oncofertility specialists.

## Funding

The authors have no funding sources to report.

## Notes

Affiliations of authors: Dana-Farber Cancer Institute (PDP, ASL, AHP); Dana-Farber Cancer Institute and Boston Children’s Hospital (ALF, AMF, PEM, LRD); Brigham and Women’s Hospital (ESG); Boston Children’s Hospital and Brigham and Women’s Hospital (MRL).
